# The knowledge, perceptions and relationship behaviour of rugby and football players towards HIV infection at the University of Limpopo

**DOI:** 10.4102/curationis.v41i1.1899

**Published:** 2018-11-14

**Authors:** Indiran Govender, Kathryn Nel, Nhlanhla Banyini

**Affiliations:** 1 Department of Family Medicine and Primary Health Care, Sefako Makgatho Health Sciences University, South Africa; 2 Department of Psychology, University of Limpopo, South Africa

## Abstract

**Background:**

Sport has the capability to unite a country. To achieve winning teams, athletes have to rely on each other and often have close physical contact. Disclosure of a positive human immunodeficiency virus (HIV) status may be problematic for athletes in contact sports as they may suffer discrimination and stigmatisation which may impact their relationship behaviours. This may impact frontline nursing and medical staff dealing with on-field ‘blood’ injuries.

**Objectives:**

The purpose of this study was to determine if individuals who participate in football and rugby are aware of the risk of HIV infection in contact sports and their perceptions and reported behaviour towards HIV-positive athletes.

**Method:**

A cross-sectional survey design with a qualitative element. Quantitative data were analysed using descriptive statistics, while thematic content analysis was used to analyse qualitative data.

Non-proportional quota sampling was used for male rugby (*n* = 23) and football (30) players registered at the University of Limpopo (Turfloop campus).

**Results:**

The results supported previous research in that there are gaps in HIV knowledge. For instance, not knowing that anal sex may cause HIV infection and believing that saliva can transmit HIV and that blood transfusions are unsafe.

**Conclusion:**

Problematic findings were that a portion of the sample believed that having sex with a virgin could cure HIV and the majority of the sample believed that being ‘bewitched’ could cause HIV and acquired immunodeficiency syndrome (AIDS).

## Introduction

### Problem statement

Sport has the ability to unite a country, transcending race, language and politics (Van Heyningen 2012), and this was evident with South Africa winning the Rugby World Cup in 2004 (Labuschagne 2009). To achieve this unity, athletes have to rely on each other, and they often have close physical contact. Disclosure of a positive human immunodeficiency virus (HIV) status can therefore be particularly problematic for athletes in contact sports, as it may lead to discrimination and stigmatisation and strained relationships (Banyini 2014; Maleka 2017).

In contact sports, there is a small risk of transmitting HIV. This risk is present due to the possibility of blood exchange, especially after an injury (Centers for Disease Control and Prevention 2013). Nursing staff and paramedics are at the vanguard of the treatment of athletes (clinics, hospitals or on the field of play), and although they always take preventative measures, there is always the slight risk of needle prick or cross-contamination infection. Consequently, there is a need for the education of athletes on HIV transmission and prevention (Kondowe 2014). This type of education is often given by allied health professions such as nurses (Gitachu 2017).

### Literature review

Contact sports like rugby and football often lead to injuries (Maite, Nel & Govender 2016). Athletes usually continue participating in a game for some time after sustaining a bloody injury (Hoogenboom & Smith 2012). The level of attention after a bloody injury is not as high in amateur sports as it is in professional sport. These factors significantly increase the risk of HIV transmission when an injury occurs. In addition, there has been a debate over mandatory HIV testing and if players should be excluded from sport if they are HIV-infected^1^ (Odendal 2011). This also raised concern about HIV-negative athletes competing in contact sports against those who are HIV-positive (Banyini 2014).

The critical question concerns the consequences of a positive HIV test result with mandatory testing. The majority of experts agree that HIV infection should not exclude an athlete from participation in most sports. Routine HIV testing is therefore considered by many experts to be unjustified (Odendal 2011; South African Sports Medicine Association 2001).

Wrestling and boxing organisations have made HIV testing compulsory (McGoldrick 2012). When an HIV-infected athlete wishes to engage recreationally or competitively in sport, individualised recommendations regarding participation should be based on the clinical status of the athlete, the possible immune response to the exercise and the risk of blood exposure. Studies of HIV knowledge of university students suggest that many athletes have mistaken beliefs about the risks of contracting HIV and acquired immunodeficiency syndrome (AIDS). Despite the small chance of contracting HIV, many athletes would not want to compete against HIV-infected athletes (Banyini 2014). Interestingly, rugby players seemed to be more likely to avoid people whom they knew were HIV-infected, both on and off the field of play, than football players (Lembethe et al. 2012).

Most athletes at both amateur and professional levels of sport do not give any thought to the possibility of contracting HIV while pursuing a healthy activity (Grosset-Janin, Nicolas & Saraux 2012). Although contact sports have a small risk of HIV infection, the main pathways of transmission of HIV in athletes are similar to those in the general population (Schöffl, Morrison & Küpper 2011).

There is evidence that moderate exercise may have psychological and immunological benefits for a patient with HIV (Nel & Tshikovhele 2018). Moreover, regular exercise improves the general sense of well-being of HIV-positive persons.

HIV is found in all bodily fluids, and transmission is known to occur only through the exchange of blood, semen and vaginal and cervical secretions. The three modes of transmission that account for most cases of HIV infection are sexual transmission, blood-to-blood transmission and vertical transmission from a mother to a foetus (health24 2014). Other modes of transmission could therefore be described as myths or misconceptions of HIV transmission.

Misconceptions and myths refer to concepts and ideas held or believed by a group of people, which are proven to be incorrect (Sano et al. 2016). The strongest predictor of discrimination and stigmatisation towards people with HIV were misconceptions about the risk of HIV transmission. Knowledge about the true main routes of HIV transmission was, however, not associated with these attitudes (Qian et al. 2007); therefore, clinical knowledge about HIV transmission is critical in eliminating such misconceptions (Maleka 2017).

Misconceptions about the transmission of HIV still exist widely (Fraim 2012), and are especially prevalent in sub-Saharan Africa. These misconceptions are present despite efforts to improve public awareness and knowledge about the disease, and are influenced by societal and individual factors, and ethnicity, region and religion also play a role in misconceptions held (Sano et al. 2016).

Some of the fallacies described in the literature are that HIV and AIDS is transmitted through social activities, or through air, rubbish or having sexual intercourse with animals. There are those individuals who see HIV infection as a punishment from God, or believe that transmission can take place through mosquito bites, ants and flies (Fraim 2012; Sano et al. 2016).

Other mistaken beliefs are directly related to contact with a person living with HIV or AIDS (PLWHA) and include having everyday contact, sharing food utensils, sharing toilet seats, wearing an infected person’s clothes, hugging, kissing or holding an infected person, swimming with an infected person or being exposed to an infected person who spits or coughs (Fraim 2012).

In relation to sports, there is a misconception that a person who is well nourished and engaged in sports could not be infected by HIV and AIDS (Fraim 2012), and that a healthy-looking person cannot be HIV-positive (Banyini 2014).

In addition, there is a belief that married people cannot be infected with HIV, even if they engage in extramarital sex, and if HIV-infected people use prescribed medicine, they will be cured completely (Fraim 2012). Other myths regarding the cure of HIV are that eating fresh vegetables, sleeping with a virgin, or making ancestral sacrifices will cure the disease (Sano et al. 2016).

Stigmatising attitudes towards persons with AIDS (PWAs) reduce athletes’ willingness to be tested for HIV (Peltzer et al. 2009). This may also influence their relationships with other athletes and significant others. In addition, the most significant barriers to HIV testing are the fear of being known by counsellors, fear of being HIV-positive, and stigmatisation by fellow athletes resulting in stressed peer relationships (Kanyemba 2010; Nqojane et al. 2012). Another reason given for not testing is the belief that a positive result means an immediate death sentence (O’Hara 2007).

According to Maleka (2017), sport is an effective platform to increase HIV and AIDS knowledge and awareness. The use of peers in sport context HIV prevention education programmes is very effective in conveying knowledge (Maro, Roberts & Sørensen 2009). Male athletes are more likely to take sexual risks than females and non-athletes. HIV prevention programmes need to be effective and aimed at high-risk groups. It is likely that sport can play an important role in HIV knowledge and behaviours.

Banyini (2014) reports that intimacy versus isolation is related to young adults wanting to enter into romantic and peer relationships. The author further notes that those who are rejected from these friendships may well experience isolation. Discrimination and isolation, because of fear of another person being infected with HIV and AIDS, may cause this type of isolation and rejection in a sporting environment.

This research aimed to determine the attitudes, knowledge and how relationships are affected relating to HIV and AIDS in rugby and football playing students at the University of Limpopo (Turfloop campus).

## Methodology

### Research design

This was a cross-sectional research design with a qualitative aspect (commensurate with triangulation of data) study that was exploratory in nature.

### Population

The study population was all contact sports players at the University of Limpopo (Turfloop Campus), Limpopo province. The demographic composition of the contact sport players at the university is black Africans, mostly from disadvantaged backgrounds.

### Sampling

Non-proportional quota sampling, the non-parametric equivalent of stratified random sampling, was used (Terre Blanche, Durrheim & Painter 2009). This method is useful as it allows a sample to be selected based on variables necessary for a study (Morrow et al. 2007). These variables included gender (males), age (18–24 years) and the nature of the sport (i.e. a designated contact sport). There were approximately 55 male rugby players and 100 football players at the time of the study. It was decided that 30 from the rugby sub-group and 50 from the football sub-group of student athletes would be suitable as this would be an appropriate balance by population proportion. However, due to non-response to questionnaires the final sample consisted of 23 rugby players and 30 football players. It was decided to use an all-male sample, as the level of female participation in contact sport at the university is very low.

### Survey questionnaire

The questionnaire was adapted from Germanos (2006), and is self-report in nature. It had a demographic section and questions related to HIV knowledge, attitudes associated with HIV and sport and HIV prevention. Section 5 consisted of open-ended questions. Information such as general HIV and AIDS knowledge, mode of transmission, prevention and treatment, and knowledge in relation to sport were evaluated.

The questionnaire has been used in other contexts (Bandawe & Foster 1996; Ndeki et al. 1994; Pattullo et al., 1994; Umeh 1997). Most of these studies were in different contexts; however, Umeh’s (1996) was conducted in a sporting environment. The survey was adapted, and additional open-ended questions were used to gain a comprehensive understanding of the athletes’ insight into the phenomena. It was pretested on four non-participating athletes and any problematic questions were corrected or removed. The final questionnaire had a high internal consistency (Cronbach’s alpha = 0.88).

The open-ended questions were as follows:

- What are your feelings and ideas about HIV prevention measures for athletes?
- What are your thoughts about HIV education and general awareness programmes and campaigns for athletes?
- How do you feel about HIV-infected athletes participating in sporting activities?
- What are your feelings about the medical assistance that HIV-infected athletes receive?
- Explain whether HIV-positive athletes should inform their coach and other athletes of their status.
- What are your feelings about discrimination towards HIV-infected athletes?
- If you or other players became infected with HIV, how would you feel about you or them continuing to participate in sport? Explain.
- Explain whether you are concerned about contracting HIV during sports participation.
- What do you think about mandatory HIV testing in sport?

### Data analysis

Quantitative data were analysed using descriptive statistics (frequencies and percentages). For the purposes of this research, good knowledge was standardised as getting 50% or more of the knowledge questions correct, and poor knowledge when the participant scored below 50%.

Thematic content analysis was used to analyse the qualitative data obtained from the open-ended questions (Terre Blanche et al. 2009). Familiarisation and immersion were achieved by reading the text thoroughly so that meanings and interpretations could be made. The result of this phase was codes and detailed notes. The next step was inducing themes out of the data. Data reduction was done to create categories for more efficient analysis. Coding was done to describe the meaning of the themes. The researchers then looked at how the themes support the data and the overarching theoretical perspectives (elaboration). Interpretation and checking were then done by defining each theme, aspects of data that were captured, and what was interesting about the themes. The data were analysed using colour coding.

Erikson’s (1956) psychosocial theory was also used to underpin the study by linking the need for relationships to the age of the athletes (20–39 years = the need for intimacy rather than isolation). As noted in this age range, there is a need for close interpersonal relationships and not isolation which may occur because of HIV and AIDS infection (Nqojane et al. 2012).

### Ethical considerations

Informed consent was obtained from all participants. Ethical clearance was obtained from the University of Limpopo (Reference number: MREC/M/205/2010:PG).

## Results

### Demographic information

The mean age of the athletes was 22.1 years (*SD* = 1.708). The majority (42, 79%) were single, 2 (3.8%) were married, 5 (9.4%) lived with partners and 4 (7.5%) were in steady relationships. Twenty-three (43.4%) played football and 23 (56.6%) played rugby. The overwhelming majority, 48 (90.6%) of the respondents, played their respective sports at an amateur level, 2 (3.8%) had played at provincial level and 2 (3.8%) had participated at national level. Only 1 (1.9%) respondent did not state at what level he had participated.

The qualitative data were obtained from open-ended questions on the questionnaire which were answered by respondents.

### Emerging themes

The themes are summarised in Table 1 and are drawn from the concepts in the open-ended questions in the survey, namely HIV testing, disclosing HIV test results, sports participation by HIV-positive athletes and coaches, attitudes towards HIV-positive athletes, prevention of HIV transmission on the sports field, education about HIV and AIDS, effect of HIV and AIDS on relationships and sport and HIV prevention. The themes are then discussed.

**TABLE 1:** Themes that emerged from the qualitative data.

Theme	Supporting quotes
Theme 1: Prevention	‘There should be prevention! Better precautions by doctors lead to better prevention of the virus spreading through the team. I don’t want to get HIV because I want a girlfriend. I don’t have a problem testing for HIV. I do it every six months since I’m sexually active. It helps me know my status so I can be able to take care of myself’ (Participant 2, Northern Sotho, Rugby player).
Theme 2: Awareness	‘The more people know about the dangers and general effects of HIV, the better the attitudes towards HIV and the infected people, so it is good to have programmes. Education programmes raise awareness amongst sport personnel on what HIV really is and ways to prevent it’ (Participant 24, Southern Sotho, Football player).
Theme 3: Well-being	‘They should not prevent people with HIV to participate in sports as it is good for their health and would keep them physically fit and help their mind. You spend a lot of time participating and trying to improve which keeps you occupied rather than taking your time looking for too many sexual activities. It is then better to have one girlfriend and keep healthy’ (Participant 16, Ndebele, Rugby player).
Theme 4: Medical assistance	‘I think there should be mandatory HIV testing for athletes because medics can then know how to treat you. There are always medics before the game starts, the game will not start without their presence. This is good because they know how to take care of blood injuries … [*but*] I don’t think the paramedics that assist know enough. Players often return to the field and bleed through the bandage’ (Participant 34, Xhosa, Football player).
Theme 5: Confidentiality	‘No, it must not be because HIV testing is personal. It is someone’s privacy, and in football it is unnecessary because no open wound is allowed. Players must cover his wound so it’s unnecessary. It is only necessary to tell the coach so he knows not to push too hard and he must not tell others’ (Participant 33, Venda, Rugby player).
Theme 6: Discrimination	‘I don’t feel comfortable [*revealing my status*] as I don’t know how others will take it and also I fear discrimination. No it would be discrimination towards that player [*and*] I wouldn’t discriminate against such a player as I know for a fact that he needs our support’ (Participant 46, Northern Sotho, Football player).
Theme 7: Fear and anxiety	‘It is scary, no matter how many times I hear about it [*HIV testing*] or am advised to do it. It makes me very scared and anxious. I will fear the person who is HIV-positive in my team, because there is a high risk of HIV infection when you play the contact sport with a person who is diagnosed with HIV’ (Participant 1, Northern Sotho, Rugby player).
Theme 8: Emotional support	‘An HIV-positive person needs exercise and support in dealing with the pandemic. All those are found in sport participation. Of course it takes guts to disclose but with professional help and support from friends and family I will be strong enough to’ (Participant 3, Venda, Football player).
Theme 9: Strategy of the sport	‘I feel pity for them and I think the strategy of full contact should change and they should play touch rugby as not much contact is allowed. Yes, there must be a change because it must be made certain that any contact does not lead to bloodshed and contact must be reduced with any other player. [*I would be*] scared, if no tactical change, because I can also be affected when we contact…[*but*] there is nothing wrong with them, so I don’t think it would be necessary to change the schedule/strategy’ (Participant 8, Northern Sotho, Rugby player).
Theme 10: Participation	‘I don’t think participating prevents or changes you in any way. I think it just takes a certain set of mind to be aware and have a healthy lifestyle. It will have no effect participating in sport as it does not have anything to do with HIV. By keeping players busy and occupied, most football players who play in sport have sex less because of the hard training regime they encounter during sport participation. This is good as, in that way, sport participation can help reduce HIV infection’ (Participant 21, Venda, Football player).
Theme 11: Mandatory testing	‘In rugby, I strongly suggest that there should … Yes, there should be mandatory HIV testing in the team so that we can all know our status and we can also encourage each other to take medication if one is affected, because if we don’t know our status that’s when someone is going to infect others’ (Participant 50, Northern Sotho, Rugby player).
Theme 12: Risk of HIV infection	‘It’s going to be challenging considering the fact that my health is at risk, in a way. I would not share the same equipment, it’s too risky. [*But*] I think it depends on the kind of sport. If it’s a fighting or contact sport yes, I can have a problem’ (Participant 3, Northern Sotho, Football player).

## Discussion

### Discussion of quantitative results

A small percentage (9.4%) of the respondents said that having unprotected sex with several people does not make one more susceptible to contracting HIV. This is alarming as it could imply that these respondents were likely to have unprotected sex with more than one partner which is supported in research by Nqojane et al. (2012) and Fraim (2012). Almost 36% of the respondents indicated that HIV cannot be transmitted through anal sex, and more than one-third were not aware that HIV could be transmitted this way. This is also problematic as it is untrue and it is a risky sexual activity. Although 92.5% knew that sharing a towel or cup could not transmit HIV, 7.5% believed it possible, while 28.3% thought that HIV could be transmitted through mosquito bites. Forty-eight (90.6%) of the athletes responded that people can acquire HIV and AIDS from being bewitched (Banyini 2014).

Forty-three respondents (81.1%) stated that homosexuals are not responsible for spreading HIV. This indicates that there is a good level of knowledge about how HIV is spread and homosexuality as has been reported in other research (Germanos 2006; Nqojane et al. 2012). It is, however, worrying that a substantial number of athletes are misinformed on this issue as it could lead to discrimination and stigmatisation.

Twenty-six (49.1%) of the athletes believed that receiving a blood transfusion in South Africa is unsafe. This belief could lead to unwillingness of athletes to consent to life-saving blood transfusions, despite strict screening measures that make blood transfusions safe in South Africa (health24 2016). The majority (77.4%) of the respondents were aware that healthier lifestyle practices are necessary to reduce the risk of contracting HIV (Sano et al. 2016). However, 36 (68%) of the athletes wrongly believed that keeping in good physical shape is the best way to prevent HIV infection.

Twenty-one (39.6%) of the athletes indicated that Western medicine does not have a cure for AIDS or that it is expensive and not accessible to them as found in previous research (Batisai 2016). However, 41 (77.4%) of the athletes believed that traditional African medicine has a cure for AIDS. This is worrying as it may lead individuals to think that AIDS is curable and not a chronic and life-threatening illness.

A concerning finding was that the majority of the athletes (86.8%) believed that having sex with a virgin can cure HIV. This misconception could promote forced sex or rape (Leclerc-Madlala 2002), and could pose a danger on campus, particularly to first-year females just entering university, who are often presumed to be virgins.

Most of the athletes (88.7%) knew that HIV cannot be transmitted through sharing sports equipment (health24 2016), but more than a third thought that HIV transmission occurs commonly during sporting activities. This is incorrect and, as has been noted by other research, indicates that athletes need HIV and AIDS education (Sano et al. 2016).

Although the majority of the athletes (98%) are aware that HIV can be transmitted through contact with blood from an HIV-infected player and 94% agreed that HIV can be transmitted through sharing needles, which is supported by Maro et al. (2009), some athletes (21%), nonetheless, indicated that a person playing sport can get HIV through perspiration/sweat which is incorrect (health24 2016).

The results of knowledge testing are presented in Table 2. Overall, 64% of the participants had good knowledge about HIV and AIDS; however, over a third did not. These results support similar findings (Nqojane et al. 2012) which suggest that there are still some gaps in knowledge with regard to HIV and AIDS in a student sample in South Africa.

**TABLE 2:** Overall HIV and/or AIDS knowledge.

HIV and AIDS knowledge	Frequency (*n*)	Percentage (%)
Poor knowledge	19	35.8
Good knowledge	34	64.2
Total	53	100.0

### Discussion of qualitative analysis

Participants indicated that there should be adequate prevention against HIV infection that was seen as an important factor in terms of intimacy, both in personal and team relationships. However, it might also lead to a discriminatory attitude towards those with HIV as the athletes stated that they did not want friendships or relationships with those who are infected. Literature suggests that HIV-related stigmatisation and discrimination is a barrier to accessing voluntary counselling and testing for HIV (VCT) and to the effectiveness of prevention and care services (Nqojane et al. 2012). In this study it was also found that those who reported stigmatising attitudes had never been tested for HIV, which supports findings in previous research (Genberg et al. 2009). However, some of the athletes stated that they were willing to go for VCT as they thought this was a good preventative measure.

It is very important for athletes to be aware of HIV and how sexual behaviours and other behaviours such as touching wounds with bare hands can impact their health and thus their personal lives (Nqojane et al. 2012). This will, in turn, assist them to make informed decisions regarding their health.

It was seen that individuals who perceive themselves as more vulnerable to HIV and AIDS are less likely to believe misconceptions about disease transmission. This is mainly because such individuals are more likely to seek more information about the disease (Sano et al. 2016), and therefore have more awareness about risks and behaviours that promote HIV transmission.

The sample of rugby and football players valued well-being and indicated that participation in sport is important for good health (health24 2016). Many indicated that knowing their HIV status would also add to well-being. However, some participants expressed fear and thought they might be discriminated against (Kanyemba 2010; Ulasi et al. 2008). This suggests that the respondents perceived that HIV-positive persons should take part in sport to have a positive and healthy sense of self or well-being. Athletes also indicated that it is better to be aware of one’s HIV status, as this will allow individuals to take better care of themselves.

Athletes indicated that HIV-positive individuals should continue playing sports for the sake of fitness and becoming good leaders (Holt et al. 2012). There are numerous positive developmental indicators that have been associated with sport participation, including improved self-esteem, positive emotional regulation, good problem-solving skills, goal attainment, positive social skills and improved academic performance. A contrasting view is that youth involved in sport can be aggressive and are prone to inappropriate behaviours, particularly older adolescent male athletes. For this reason, participation in sport will not automatically result in healthy lifestyle choices (Banyini 2014).

Respondents felt that if the medical team is aware of an athlete’s positive HIV status, they can take appropriate precautionary measures. They also said that sports administrators, coaches and managers should ensure that adequate medical care is available when athletes participate in sport (Banyini 2014). Rugby and football players who participated in the research were confident that the medical attention that they received was good enough; however, there were some who were dissatisfied with their medical assistance. These athletes stated that paramedics and nurses did not take adequate precautions regarding cross-infection when dealing with blood injuries.

Although many participants felt that an individual’s HIV status is confidential, some felt that mandatory testing should be in place (which is suggested by the South African Sports Medicine Association 2001) as they would not feel comfortable playing in a team with an HIV-positive individual. Some respondents maintained that the coaching and medical staff should be informed about any HIV-positive athlete, whereas others responded that coaching and medical staff should not be informed about the HIV status of athletes as that is a ‘personal choice’. This is not in line with many sports as, for instance, Cycling South Africa (2010) stipulates that an athlete should inform the coach if they are HIV-infected so that appropriate interventions can be made in the event of an injury. The policy further states that the individual should be treated in a calm, open, honest way. Overall, many of the rugby and football players are not in favour of letting other athletes know. This suggests that some of the respondents may fear discrimination and stigmatisation by their teammates, coaching and medical staff (Germanos 2006). Nonetheless, it remains the privilege of the infected athlete to disclose information related to this particular aspect of their medical history. However, it should be noted that adherence to such a policy may aid in the prevention of discrimination and stigmatisation against HIV-positive athletes.

Health-related stigma is defined as:

a social process, experienced or anticipated, characterised by exclusion, rejection, blame or devaluation that results from experience, perception or reasonable anticipation of an adverse social judgement about a person or group. (Genberg et al. 2009:2)

AIDS-related stigma is defined as: ‘the prejudice, discounting, discrediting and discrimination that are directed at people perceived to have AIDS’ (Qian et al. 2007:1).

HIV infection carries immense stigma and discrimination (Germanos 2006). The results of this study infer that athletes are afraid of being discriminated against because familiar relationships play a pivotal role in their development (Erikson 1956). Athletes fear discrimination as they need to maintain relationships with their team members and fear that if they are known to be HIV-positive, these relationships will be lost. However, there were participants who reported that they would not discriminate against an HIV-positive athlete.

Respondents fear the implications of a positive result. It was also reported that they fear playing with, or against, athletes who are HIV-positive. Unfortunately, this suggests that stigmatising attitudes towards PWAs reduce their willingness, and increase their fear and anxiety, of being tested (Pelzer, Nzewi & Mohan 2004). Other researchers have found that the most significant barriers to VCT services are the fear of being known (by counsellors), fear of being HIV-positive and stigmatisation (Kanyemba 2010; Nqojane et al. 2012). Another reason, found in this research, for not testing is the fear that a positive result means an immediate death sentence which is supported by previous findings (O’Hara 2007).

Athletes suggest that emotional support is needed for an HIV-positive athlete. This support should come from family members, friends and teammates. High levels of discrimination against HIV-infected individuals have been seen, where poor care and support were available. Informal communication about HIV and AIDS was associated with lower HIV-related stigma, and more negative attitudes were held towards HIV-infected people by individuals who had never talked about HIV and AIDS with anyone. Informal discussion about HIV and AIDS with family members, friends or teammates could result in greater knowledge of HIV and AIDS, decrease HIV-related discrimination and even alter high-risk behaviours related to HIV (Genberg et al. 2009).

Respondents also suggested that strategy on the sports field should relate to avoiding full contact, which implies that rules may need changing in sports like rugby which was also noted in research by Banyini (2014). Participants felt that HIV and AIDS awareness programmes helped them make informed decisions pertaining to their lifestyles. Participants indicated empathy for HIV-positive players but, at the same time, feel that the strategy or tactics in their sports should be revised when HIV-positive athletes participate. Fundamentally, this demonstrates discriminatory attitudes as non-acceptance of HIV-positive players, and is likely to lead to their stigmatisation both on and off the field.

Participants felt that taking part in sports has no effect on HIV infection. They also indicated that everyone has the right to participate in any kind of sport, and were not concerned because HIV transmission through sport is very rare which implied they were not discriminatory or were ‘saying the right thing’. Nonetheless, there is a lack of evidence to indicate that sport-based HIV transmission is a problem (Delva et al. 2010). In fact, the risk of being infected with HIV during any sporting activity is extremely low (Banyini 2014). This supports the above statements. Some studies indicate that sport has a positive effect on HIV prevention (Kaufman, Spencer & Ross 2012), and this is also echoed in our study.

This reflects the need by individuals of this age to forge intimate friendships with others through teamwork which is in line with developmental norms in this age range (Erikson 1956). Team friendships are the opposite of athletes who prefer sports which isolate them from others (i.e. tennis). The responses may infer that some athletes felt that keeping busy means less time for unhealthy behaviours. For instance, the comment that athletes ‘have less sex because of the need for hard training’ suggests that intimate relationships may not be entered into by some athletes because of fear of HIV and AIDS.

Current health status should guide the decision to allow continued participation of an athlete with HIV and AIDS or HIV-related infections, and should be according to the discretion of the athlete’s personal physician, in addition to the athlete himself or herself. The same standards and procedures used for all athletes should be used to determine the eligibility to participate in athletics of an HIV-infected athlete. To ensure the best interests of the HIV-infected athlete, regular medical check-ups are recommended (Maleka 2017).

Rugby and football players in this research indicated that mandatory testing was necessary; however, they are unclear as to how this would be of benefit to players as a whole, as many had previously noted that knowledge related to VCT was confidential. However, mandatory testing is difficult as some young adults may not be ready to test. Studies show that students may become depressed and feel despair at an HIV-positive result (Maleka 2017; Nqojane et al. 2012), and some athletes might feel that they do not want to participate in sport for fear of transmitting HIV.

Although mandatory testing could be a way of preventing the spread of HIV through sport participation, this testing will have important consequences. The first is that an athlete who tests positive would be able to enter into a treatment programme early, thereby benefiting from the test. A second consequence, however, is that such an athlete may be prohibited from participating in sport, and be stigmatised and discriminated against (Banyini 2014). HIV infection alone is insufficient grounds to prohibit athletes from participating in sport. Mandatory HIV testing for athletes is not justified in literature, mainly due to the low risk of HIV transmission in sport participation (Maleka 2017).

There was a perception that there is a risk involved in playing contact sports when an individual is HIV-positive, although the risk of HIV transmission through sport is very low (Grosset-Janin et al. 2012). The participants who thought that there is a high risk of transmission stated that they would probably isolate themselves if they were HIV-positive or knew of HIV-infected teammates.

The qualitative data supported the quantitative information in that the sample of athletes revealed gaps in knowledge that suggested their anxiety about the possibility of HIV infection on the field of play (a very remote risk). There is stigma attached to HIV, and although the sample suggested emotional support for any HIV-infected athletes, they reported that they would isolate themselves from any infected team members they were aware of. This relates to quantitative findings in terms of erroneous beliefs and the fact that over a third of the sample thought that HIV transmission can happen during a sporting endeavour.

### Recommendations

A larger study should be conducted across more contact sporting codes using a random sample. A similar study should be conducted amongst non-contact sport players to see if findings are comparable.

## Conclusion

Gaps in respondents’ knowledge relate to results that infer a portion of the sample does not know that anal sex can result in HIV infection, who think that saliva can transmit HIV infection, believe that blood transfusions are unsafe, think that traditional medicine has a cure for the disease and that many cases of HIV transmission occur through sports injuries. Other gaps in HIV knowledge include the belief that keeping in good physical shape is the best way to stop becoming infected with the retrovirus, whereas nearly 50% of the sample believe that being bewitched results in HIV infection. Some respondents believed that having unprotected sex with several different partners does not make an individual more vulnerable to HIV infection. A small percentage of the sample also thought that mosquito bites, cuts and sweat are also responsible for the transmission of HIV. The number of respondents who believe that having sex with a virgin is a cure for HIV and AIDS was worrying as was the finding that some respondents felt that homosexuals are responsible for spreading the retrovirus. The latter two findings may cause hate or sex abuse crimes. Over 60% of the sample had good HIV knowledge but, in 2018, it is worrying that over a third of respondents (in this sample) still displayed poor knowledge.

The open-ended questions gleaned knowledge which revealed respondents value well-being and indicated that participation in sport is important for good health and many indicated that knowing one’s HIV status would also add to well-being. However, some participants expressed fear and issues with discrimination. Many participants posited that an individual’s HIV status is a private matter thus confidential; however, others felt that mandatory testing should be in place and that they would not feel comfortable playing in a team with an HIV-positive individual (which suggests discrimination). This, despite the fact that some participants noted medical assistance was adequate. Conversely, some respondents felt that medical assistance was inadequate. Strategy on the sport field should relate to avoiding full contact which implies that in sports like rugby rules may need changing. Participants felt that it was a risk to their health competing or playing sport with individuals who were HIV-positive. Generally, the sample felt that emotional support from family, friends, team management and staff was needed for HIV-positive athletes. It is also inferred by participants’ responses that peer and intimate relationships are affected by fear of HIV and AIDS transmission.

The results show that there are still some gaps in knowledge with regard to HIV and AIDS amongst a portion of the sample. Broadly, this implies that amongst this sample there are still some misconceptions pertaining to HIV and AIDS knowledge. The qualitative results suggest that these gaps in knowledge may well result in discriminatory behaviour by some of the participants.

## Footnotes

1 The author acknowledges and understands the sensitive nature of terms used in the article such as ‘HIV-infected’. As the terms have been used during the research and in the documentation, e.g. the questionnaires, it has been used throughout the article.
